# Abnormal Structure–Function Coupling in Major Depressive Disorder Patients With and Without Anhedonia

**DOI:** 10.1155/da/1925158

**Published:** 2025-03-20

**Authors:** Qingli Mu, Congchong Wu, Yue Chen, Yuwei Xu, Kejing Zhang, Ce Zhu, Shaohua Hu, Manli Huang, Peng Zhang, Dong Cui, Shaojia Lu

**Affiliations:** ^1^Department of Psychiatry, The First Affiliated Hospital, Zhejiang University School of Medicine, Zhejiang Key Laboratory of Precision Psychiatry, Zhejiang Engineering Center for Mathematical Mental Health, Hangzhou, Zhejiang, China; ^2^Faculty of Clinical Medicine, Zhejiang University School of Medicine, Hangzhou, Zhejiang, China; ^3^Department of Psychiatry, Jinhua Municipal Central Hospital, Jinhua, Zhejiang, China; ^4^Department of Psychiatry, Zhejiang Xiaoshan Hospital, Hangzhou, Zhejiang, China; ^5^School of Radiology, Shandong First Medical University and Shandong Academy of Medical Sciences, Tai'an, China

**Keywords:** anhedonia, frontal lobe, major depressive disorder, ROC, structure–function coupling, temporal lobe, thalamus

## Abstract

**Background:** As a core symptom of major depressive disorder (MDD), previous magnetic resonance studies have demonstrated that MDD with anhedonia may exhibit distinctive brain structural and functional alterations. Nevertheless, the impact of anhedonia on synchronized alterations in the structure and function of brain regions in MDD remains uncertain.

**Methods:** A total of 92 individuals were enrolled in the study, including 29 MDD patients with anhedonia, 33 MDD patients without anhedonia, and 30 healthy controls (HCs). All subjects underwent structural and resting-state functional magnetic resonance imaging (MRI) scans. The structure–function coupling of cortical and subcortical regions was constructed by using the obtained structural and functional data to quantify the distributional similarity of gray matter volume (GMV) and amplitude of low-frequency fluctuations (ALFFs). Analysis of covariance (ANCOVA) was used to compare differences in structure–function coupling among the three groups. Partial correlation analyses were conducted to identify relationships between structure–function coupling and clinical features. Finally, receiver operating characteristic (ROC) curve and support vector machine (SVM) analysis were employed to verify the capacity to distinguish between MDD with anhedonia and MDD without anhedonia, MDD with anhedonia and HCs, and MDD without anhedonia and HCs.

**Results:** The ANCOVA revealed significant differences in structure-function coupling among three groups in the bilateral precentral gyrus (PrG), right insular gyrus (INS), right cingulate gyrus (CG), right thalamus (Tha), left superior temporal gyrus (STG), and left middle temporal gyrus (MTG). Compared to HCs, both MDD groups showed reduced coupling in the right INS, bilateral PrG, while increased coupling in the right CG. Additionally, MDD with anhedonia showed reduced coupling in the right Tha, right PrG, and left MTG, while increased coupling in the left STG, compared to the other two groups. Furthermore, ROC analyses indicated that structure-function coupling in the right PrG, right CG, and left MTG exhibited the greatest capacity to distinguish between the following groups: MDD with anhedonia from HCs, MDD without anhedonia from HCs, and MDD with anhedonia from MDD without anhedonia. The combined metrics demonstrated greater diagnostic value in two-by-two comparisons.

**Conclusion:** The present findings highlight that altered structure–function synchrony in the frontal, temporal lobes, and Tha may be implicated in the development of symptoms of anhedonia in MDD patients. Altered structure–function coupling in the aforementioned brain regions may serve as a novel neuroimaging biomarker for MDD with anhedonia.

## 1. Introduction

Anhedonia is regarded as a core diagnostic feature of major depressive disorder (MDD). This refers to the diminished capacity to enjoy pleasure in most or even all activities [1]. Studies indicate that ~70% of MDD patients experience anhedonia [2]. In clinical terms, patients exhibiting pronounced anhedonia symptoms tend to present with more severe symptoms and a higher risk of self-injury and suicide throughout the course of the disease [3, 4]. It is notable that anhedonia typically persists following the improvement of other symptoms, seriously affecting the recovery of social functioning [5–7]. Therefore, anhedonia is also considered a prognostic factor in MDD patients [8–10]. Similarly, during the course of MDD treatment, patients with anhedonia exhibit inferior therapeutic outcomes and a reduced reliance on therapy, both of which interact and reinforce one another [11, 12]. A growing body of research supports the recognition of anhedonia as a more severe subtype of MDD [13, 14]. Therefore, a comprehensive understanding of the biological mechanism of MDD with anhedonia is of great guiding significance for clinical treatment.

Nowadays, multimodal magnetic resonance imaging (MRI) technology has provided important insights into the structural and functional abnormalities of MDD with anhedonia. For functional changes in MDD with anhedonia, the amplitude low-frequency fluctuation (ALFF) index based on resting-state MRI has been repeatedly reported to reflect the abnormal spontaneous neuronal activity in MDD patients with anhedonia. A recent study revealed that, compared with MDD patients with low anhedonia, the ALFF values in high anhedonia patients were increased in the right fusiform, left inferior frontal gyrus (IFG), pars orbitalis, and left inferior temporal gyrus (ITG), while the ALFF values of the above brain regions tend to be normal when anhedonia was alleviated [15]. In addition, the ALFF values of the anterior cingulate cortex (ACC) and orbitofrontal cortex (OFC) have also been proposed as potential imaging biomarkers for MDD with anhedonia [16]. Regarding structural changes in MDD with anhedonia, a host of previous research has indicated potential associations between MDD with anhedonia and alterations in gray matter volume (GMV) in brain regions involved in reward circuits, such as the caudate [17, 18] and nucleus accumbens (NAcc) [19, 20]. More interestingly, previous studies have indicated that reduced cortical blood flow was associated with brain atrophy, and brain region function was dependent on cerebral blood flow [21]. Evidence from longitudinal studies showed that as symptoms of depression resolved, there was a gradual increase in both volume and blood flow [22, 23].

Convergent evidence has revealed that brain structure and function are inherently intertwined and coupled [24]. That is, alterations in brain structure impact the function of the corresponding region, and abnormalities in primary function may result in secondary volume alterations [25]. However, previous research on MDD with anhedonia has often employed neuroimaging data of different modes for independent analysis and comparison, thereby ignoring the potential for interactions between brain structure and function, as well as the underlying neuropathology. Emerging studies have indicated aberrant brain structure–function interactions in the pathology of psychiatric disorders, including bipolar disorder (BD) [26–28], schizophrenia (SZ) [29], and attention deficit hyperactivity disorder (ADHD) [30]. In a recent cross-sectional study of 168 participants with adolescent MDD and 101 healthy controls (HCs), common and unique structural and functional connectivity coupling changes were identified in the hub regions of the default mode network (DMN), visual network, and frontal-limbic circuit [31].

Taken together, the primary goal of the present study was to examine the alterations of structure-function coupling in MDD by integrating both structural MRI and resting-state fMRI data. For structural MRI, the study calculated the GMV to respond to the structural alterations of the whole brain, which is a commonly used metric for the exploration of neurobiological mechanisms of psychiatric disorders and can directly respond to the size of the volume of the brain regions with high stability of measurement. In addition, recent studies have successfully correlated GMV with data on gene expression and peripheral inflammatory markers, confirming its high comparability in cross-omics data [32]. For resting-state fMRI, ALFF analysis was chosen to respond to whole-brain functional changes. The ALFF is a quantitative measure of the regional intensity of neuronal spontaneous fluctuations during the blood oxygen level-dependent (BOLD) time course to quantify the autonomic activity of localized brain regions, which has the advantage of eliminating the need to preset seed points or network templates and avoiding selection bias introduced by a priori assumptions. In light of previous research revealed that MDD subtypes of anhedonia exhibit unique brain abnormalities in GMV and ALFF, we hypothesized that GMV–ALFF coupling would serve as a reliable neuroimaging parameter to reflect subtle brain abnormalities in MDD with and without anhedonia and that these abnormal brain regions might be utilized separately or in combination as a biomarker to assist in the diagnosis of MDD and MDD with anhedonia. This study aimed to provide a novel insight into the neurobiological basis of MDD with anhedonia by integrating multimodal brain images.

## 2. Methods

### 2.1. Participants

Patients aged 18–45 years with MDD (*n* = 62) and who were first-episode drug-naive and/or recurrent depression with continued withdrawal for more than 3 months were recruited from the Department of Psychiatry, The First Affiliated Hospital, Zhejiang University School of Medicine in Hangzhou, China. Meanwhile, sex- and age-matched HCs (*n* = 30) were recruited from local residents, hospital staff, and students through the online advertisement. In accordance with Item 2 (loss of interest or pleasure) of the symptom criteria (A) for MDD in the Diagnostic and Statistical Manual of Mental Disorders, IV Edition (DSM-IV), MDD patients were divided into MDD with anhedonia (*n* = 29) and MDD without anhedonia (*n* = 33) group.

The detailed inclusion criteria for MDD patients were as follows: (1) total score of the 17-item Hamilton Depression Scale (HAMD-17, score range: 0–34, with higher scores indicating greater severe depression symptoms) [33] ≥17; (2) right-handedness; (3) could follow the instructions to keep still during MRI scanning. The general exclusion criteria for all individuals included: (1) existence of any major medical disease; (2) current use of any medication that might affect the central nervous system, (3) drug or alcohol dependence or abuse; (4) female with pregnancy; (5) contraindications to MRI scanning, including retractors or braces, metallic implants, and claustrophobia; (6) with histories of electroconvulsive therapy (ECT). A comprehensive medical history was obtained for each participant, and a thorough physical examination was performed by the same experienced psychiatrists.

This cross-sectional study was approved by the local medical ethics committee of The First Affiliated Hospital, Zhejiang University School of Medicine. Each participant signed a written informed consent prior to the commencement of the study. This study is one of our serial investigations focusing on MDD patients, and the recruitment of participants has been described in our previous studies [34–40].

### 2.2. Assessment of Anhedonia

The Snaith–Hamilton Pleasure Scale (SHAPS) [41] was employed to evaluate anhedonia symptoms. The questionnaire is self-reported, covering four domains of hedonic experience: interests and pastimes, social interactions, sensory experiences, and diet. It comprises 14 items, each with four potential responses. The respondents were instructed to indicate their level of agreement with the statements on the scale by selecting one of four options: Strongly disagree, Disagree, Agree, or Strongly agree. For the purposes of analysis, the responses were coded as follows: “Strongly agree” and “Agree” were assigned a value of 0, while “Disagree” and “Strongly disagree” were assigned a value of 1. A total SHAPS score > 5 indicates the presence of severe anhedonia. The Chinese version of SHAPS was used in our study, and it has been shown to be a reliable and promising tool for the evaluation of anhedonia in both clinical and nonclinical populations within Chinese settings [42].

### 2.3. MRI Acquisition

Imaging data were collected using a 3.0-T scanner (Signa HDxt, GE Healthcare, USA) equipped with a standard birdcage head coil at the Magnetic Resonance Center, The First Affiliated Hospital, Zhejiang University School of Medicine. All participants were instructed to assume a supine position on the MRI scanner bed, remain still and keep awake with their eyes closed. Sagittal 3D T1-weighted images were acquired by a brain volume (BRAVO) sequence with the following parameters: TR = 7.3 ms, TE = 3.0 ms, TI = 1100 ms, flip angle = 7°, FOV = 256 × 256 mm^2^, Matrix = 256 × 256, slice thickness = 1 mm, bandwidth = 31.25 KHz, NEX = 1, slices = 192. Resting-state fMRI data were collected using a gradient echo (GRE) and echo planar imaging (EPI) sequence with the following parameters: axial slice, TR = 2000 ms, TE = 30 ms, flip angle = 90°, FOV = 220 × 220 mm^2^, Matrix = 64 × 64, slice thickness = 4.0 mm, spacing = 0.6 mm, slice order = interleaved and bottom-up, slices = 33, measurements = 180, scan time = 6.00 min.

### 2.4. MRI Data Preprocessing

For structural MRI data, the preprocessing was conducted using the Computation Anatomy Toolbox (CAT12, Christian Gaser; Department of Psychiatry, University of Jena) based on Statistical Parametric Mapping 12 (SPM 12, https://www.fil.ion.ucl.ac.uk/spm/software/spm12). The procedures were as follows: (1) Segmentation: individual structural MRI images were segmented into GM, white matter (WM), and cerebrospinal fluid (CSF); (2) Normalization: the GM images were normalized to the Montreal Neurological Institute (MNI) space using Diffeomorphic Anatomical Registration Through Exponential Lie Algebra (DARTEL); (3) Modulation: the normalized GM images were then nonlinearly modulated; (4) Resampling: the resulting GM images were resampled to 1.5 mm^3^ voxels; (5) Smoothing: The resampled GM images were spatially smoothed with a Gaussian kernel of 6 mm full width at half maximum (FWHM); (6) Output: This preprocessing resulted in a GMV map for each participant. To be supplemented was the total intracranial volume (TIV), calculated as the sum of GM, WM, and CSF volumes, as a covariate for further statistical analyses.

For the fMRI data, SPM12 and DPABI software were utilized for the preprocessing. The detailed steps were as follows: (1) Get rid of the first five time points to minimize the effect of magnetic field instability; (2) Slice timing correction was applied to eliminate the within-scan acquisition delay between slices; (3) Realignment to the first volume using a six-parameter spatial transformation to correct for head motion, excluding data with translations >2.5 mm, rotations >2.5°, or mean framewise displacement (mFD) > 0.5 [43] (*F* = 3.873, *p* = 0.0024); (4) Registration of T1-weighted images and functional images was conducted, followed by segmentation into GM, WM, and CSF; (5) The fMRI images were spatially normalized to the MNI space (1.5 × 1.5 × 1.5 mm^3^); (6) Spatial smoothing was performed on the standardized fMRI images using a Gaussian kernel of 6 mm FWHM; (7) Detrending and bandpass filtering (0.01–0.08 Hz) were applied to reduce the impact of low-frequency drift and high-frequency physiological noise; (8) Regression of covariates included 24 head motion parameters (6 head motion parameters and 6 motion parameters and squares at the previous time point), as well as WM and CSF signal.

### 2.5. Calculation of ALFF

ALFF is closely related to the time course of the BOLD signal across different regions, indicating the strength of regional brain activity. The ALFF map for each participant was calculated using preprocessed fMRI data. The time course of each voxel was converted to the frequency domain with a fast Fourier transform (FFT) to obtain the power spectrum. The square root of the power was calculated and averaged across 0.01–0.08 Hz. This averaged square root was treated as the ALFF value for each voxel [44].

### 2.6. Calculation of Structure–Function Coupling

The human Brainnetome atlas (BNA), comprising 246 regions including 210 cortical and 36 subcortical regions, served as the basis for generating regional structure–function coupling maps for each participant. The process involved: (1) Extracting GMV and ALFF values from all voxels within each brain region; (2) Separately computing probability density functions of GMV and ALFF using a normal kernel function (ksdensity in MATLAB); (3) Deriving probability distribution functions (PDFs) from these density functions; (4) Calculating the Kullback–Leibler (KL) divergence of the PDFs of GMV and ALFF within each region as follows:

  $$\begin{array}{c} D_{KL}\left(P,Q\right)=\sum_{i=1}^{n}\left(P\left(i\right)\log\frac{P\left(i\right)}{Q\left(i\right)}\log\frac{Q\left(i\right)}{P\left(i\right)}\right). \end{array}$$

(5) Transformed the KL divergence into KL divergence-based similarity (KLS) as follows:

  $$\begin{array}{c} KLS\left(P,Q\right)=e^{-D_{KL}\left(P,Q\right)}, \end{array}$$

with *e* is natural exponential. The KLS ranges from 0 to 1.

To be specific, the KL divergence is a measure of the difference between two probability distributions in probability theory, and it has been used to construct individual structural brain networks [45]. In the present study, KLS values were used to reflect the structure–function coupling of each brain region, with higher values indicating more similar distributions of GMV and ALFF. Additionally, the overall similarity of GMV and ALFF distributions across the entire brain was calculated for each participant and included as a covariate in further statistical analyses.

### 2.7. Statistical Analysis

Demographic and clinical data were analyzed using the Statistical Package for the Social Sciences (SPSS) version 27.0 (SPSS Inc., Chicago, IL, USA). Categorical variables were assessed using the chi-square test, while continuous variables were analyzed using one-way analysis of variance (ANOVA). The structure–function coupling was quantified using the KLS values for each brain region. Analysis of covariance (ANCOVA) and multiple comparisons were used to compare the differences in KLS values among the three groups. Age, gender, education years, mFD, TIV, and whole-brain coupling were included as covariates to account for potential confounding factors. The FDR correction was used to adjust the *p*-values for multiple comparisons. All tests were two-sided, with a significance level of *p* < 0.05. In addition, partial correlation analysis was performed for exploring the relationship between KLS values of significantly different brain regions obtained by ANCOVA and SHAPS scale scores and clinical characteristics (including onset age, episode times, and illness duration) in MDD patients, and Age, gender, education years, mFD, TIV, and whole-brain coupling were used as covariates. Further, FDR correction was conducted.

### 2.8. Receiver Operating Characteristic (ROC) Curve Analysis

ROC curve analyses were performed using MedCalc statistical software to extract KLS values for brain regions that exhibited significantly altered in the multiple comparisons. The ability to distinguish between MDD with anhedonia and MDD without anhedonia, MDD with anhedonia and HCs, and MDD without anhedonia and HCs was subsequently investigated by combining the above indicators with statistically significant differences. Logistic regression analysis was conducted on the KLS values of the brain regions with significant changes from multiple comparisons to calculate the prediction probability *p* (PRE), and then ROC curves were performed on the PRE. Meanwhile, the greatest Youden index (specificity + sensitivity −1) [46], as well as the corresponding specificity, sensitivity, and 95% confidence intervals (CIs), were calculated for all brain regions to summarize the overall diagnostic performance of the tests.

### 2.9. Support Vector Machine (SVM) Analysis

In order to further synthesize and validate the ability of post-hoc tests to discriminate between significantly different brain regions, this study used a linear SVM model in the Classification Learner application based on MATLAB. R 2022a version. Automated data preprocessing was designed within the Classification Learner application, which consisted of two main steps: (1) Cross-validation; in this study, the model was validated using the K-fold cross-validation (*K* = 5) method in order to avoid overfitting and to improve the stability and generalization of the models [47]; (2) Test set and training set division, this study divided the data into training set (80%) and test set (20%), the training set was used to train the model, while the test set was used to evaluate the generalization ability of the model. Finally, in terms of model evaluation, the study used several performance metrics, including accuracy, precision, recall, F1-score, AUC, and Matthews correlation coefficient (MCC), to comprehensively evaluate the classification performance of the model.

## 3. Results

### 3.1. Demographic and Clinical Characteristics

As shown in Table 1, there were no significant differences in age (*F* = 2.165, *p* = 0.121), gender (*χ*^2^ = 2.579, *p* = 0.081), or education years (*F* = 1.571, *p* = 0.214) among the three groups. No significant differences were observed between the two groups of MDD patients on means of in-onset age (*t* = 1.368, *p* = 0.177), illness duration (*t* = 0.414, *p* = 0.680), episode times (*t* = 0.868, *p* = 0.389), and the 17-HAMD scores (*t* = 1.178, *p* = 0.243). As anticipated, the MDD with anhedonia group showed higher SHAPS scores when compared to the MDD without anhedonia group (*t* = 9.642, *p* < 0.001).

### 3.2. Abnormal GMV–ALFF Coupling Among Groups

**Figure 1:**
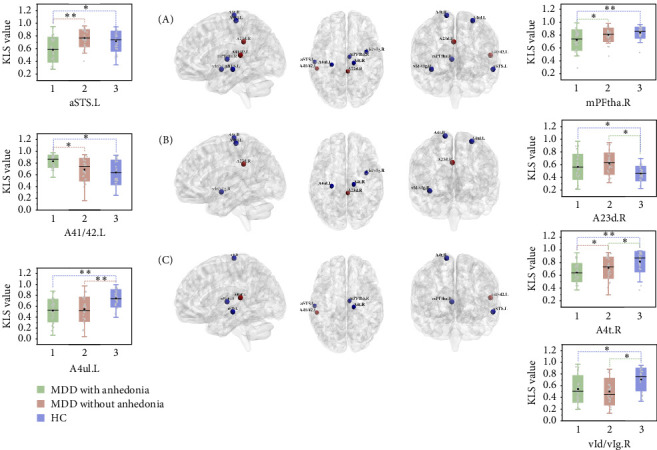
Brain regions with significantly altered GMV–ALFF coupling among three groups. Brain regions revealing distinctly abnormal GMV–ALFF coupling in (A) MDD with anhedonia and HCs; (B) MDD without anhedonia and HCs; (C) MDD with anhedonia and MDD without anhedonia. The red and blue color dots represent the level of significance for GMV–ALFF coupling increases and decreases in the CSVD-s group, respectively. A23d, dorsal area 23; A41/42, area 41/42; A4t, area 4 (trunk region); A4ul, area 4 (upper limb region); aSTS, anterior superior temporal sulcus; KLS, Kullback–Leibler divergence-based similarity; mPFtha, medial prefrontal thalamus; PRE, prediction probability *p*; vId/vIg, ventral dysgranular and granular insula.

As shown in Table 2 and Figure 1, the ANCOVA revealed significant KLS value differences among three groups in the precentral gyrus (PrG) (left: *p* < 0.001, adjusted *p* = 0.006; right: *p* < 0.001, adjusted *p* = 0.007, FDR corrected), left middle temporal gyrus (MTG) (*p* < 0.001, adjusted *p* = 0.008, FDR corrected), left superior temporal gyrus (STG) (*p* = 0.003, adjusted *p* = 0.046, FDR corrected), right insular gyrus (INS) (*p* < 0.001, adjusted *p* = 0.018, FDR corrected), right cingulate gyrus (CG) (*p* = 0.002, adjusted *p* = 0.043, FDR corrected), and right thalamus (Tha) (*p* < 0.001, adjusted *p* = 0.018, FDR corrected). As compared with HC, both groups of MDD patients showed significantly decreased KLS values in the right INS and bilateral PrG while increased KLS values in the right CG. Notably, the KLS values of MDD with anhedonia were further reduced in the left MTG, right PrG, and right Tha and increased in the left STG relative to the other two groups. In MDD patients, partial correlation analyses revealed that the KLS value of the left STG was positively correlated with the SHAPS total score (*r* = 0.44, *p* = 0.001), and the KLS value of the left MTG was negatively correlated with the illness duration (*r* = −0.29, *p* = 0.028). After FDR correction, only the KLS value of the left STG remained significantly positively correlated with the total SHAPS score.

### 3.3. ROC Curve Analysis Results

**Figure 2:**
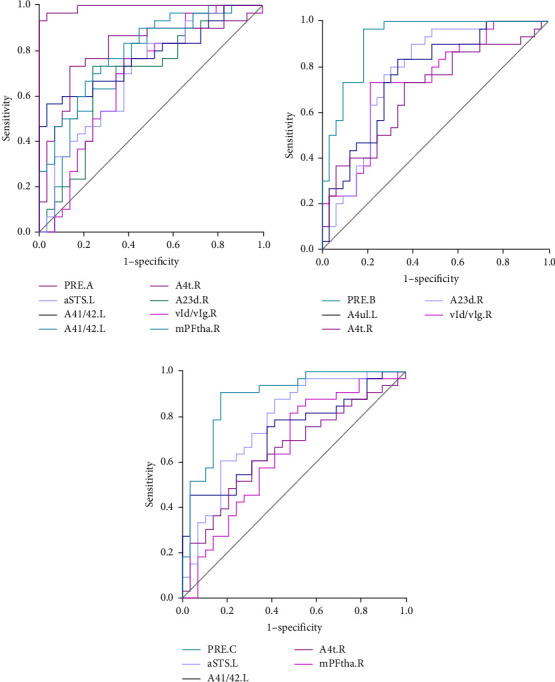
Receiver operating characteristic (ROC) curve analysis results. ROC curves for brain clusters that discriminate (A) MDD with anhedonia patients from HCs, PRE.A: AUC = 0.993, *p* < 0.001, discriminate (B) MDD without anhedonia patients from HCs, PRE.B: AUC = 0.923, *p* < 0.001, and discriminate (C) MDD with anhedonia patients from MDD without anhedonia, PRE.C: AUC = 0.866, *p* < 0.001. A23d, dorsal area 23; A41/42, area 41/42; A4t, area 4 (trunk region); A4ul, area 4 (upper limb region); aSTS, anterior superior temporal sulcus; mPFtha, medial prefrontal thalamus; PRE, prediction probability *p*; vId/vIg, ventral dysgranular and granular insula.

As shown in Table 3 and Figure 2, in terms of the Youden index, area under the ROC curve (AUC), and 95% CI for discriminating MDD with anhedonia patients from HCs, MDD without anhedonia patients from HCs, and MDD with anhedonia patients from MDD without anhedonia patients, the KLS values of the right PrG (AUC = 0.806, *p* < 0.001), right CG (AUC = 0.776, *p* < 0.001), and left MTG (AUC = 0.774, *p* < 0.001) achieved the best performance, respectively. More importantly, the combined metrics PRE (A: MDD with anhedonia and HCs: AUC = 0.993, *p* < 0.001; B: MDD without anhedonia and HCs: AUC = 0.923, *p* < 0.001; C: MDD with anhedonia and MDD without anhedonia: AUC = 0.866, *p* < 0.001) showed better discrimination performance than the individual metrics. Each significantly different brain region met the AUC significance threshold of *p* < 0.05. These brain regions, individually or in combination, have the potential to contribute as diagnostic neuroimaging biomarkers.

### 3.4. SVM Analysis Results

**Figure 3:**
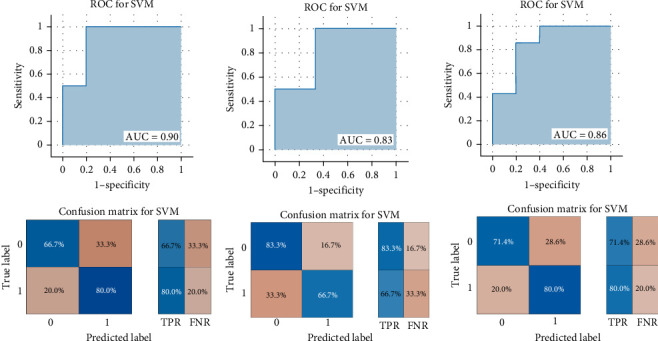
Support vector machine (SVM) analysis results. (A) MDD with anhedonia vs. HCs; (B) MDD without anhedonia vs. HCs; (C) MDD with anhedonia vs. MDD without anhedonia. AUC, area under the ROC curve; FNR, false negative rate; ROC, receiver operating characteristic; TPR, true positive rate.

As shown in Figure 3, the results of binary classification analysis of different groups (MDD with anhedonia vs. HCs, MDD without anhedonia vs. HCs, MDD with anhedonia vs. MDD without anhedonia) using the SVM model in this study showed that A: in the classification task of MDD with anhedonia patients group and HCs group, the model accuracy was 72.70%, the AUC was 0.90, the recall was 0.80, the precision was 66.67%, the F1-score was 0.73, and the MCC was 0.47; B: in the classification task of MDD without anhedonia patients group and HCs group, the model accuracy was 75.00%, the AUC was 0.83, the recall of 0.67, precision of 80.00%, F1-score was 0.73, and MCC was 0.51; C: in the classification task of MDD with anhedonia patients group and MDD without anhedonia patients group, the model accuracy was 75.00%, the AUC was 0.86, the recall was 0.80, the precision was 66.67%, and the F1-score was 0.73, and the MCC was 0.51. In comparison with the univariate ROC analysis results, the SVM model showed that the combination of multiple brain regions demonstrated a larger AUC and possessed a higher diagnostic value.

## 4. Discussions

In the current research, the KLS values were applied to reflect the GMV-ALFF coupling, thereby elucidating synchronized structural and functional alterations within the same brain region. Compared to HCs, MDD patients exhibited significantly decreased GMV–ALFF coupling in the right INS, right PrG, and left PrG, with an increase observed in the right CG. Specifically, MDD patients with anhedonia showed a marked decrease in GMV–ALFF coupling in the right PrG, left MTG, and right Tha, while an increase was noted in the left STG. Increased GMV–ALFF coupling may indicate synchronization of disruptive brain activity and morphological alterations or enhanced correlation between functional interactions and underlying anatomical structures [48]. Conversely, decoupling may suggest the opposite. These findings suggest that MDD patients with anhedonia experience distinct patterns of structure–function dysregulation. Furthermore, the study also demonstrated that abnormalities in the structure–function coupling of the aforementioned brain regions may provide new insights into the diagnosis of MDD with and without anhedonia. Overall, multimodal magnetic resonance fusion studies play a crucial role in the neurobiological mechanisms and clinical diagnosis of MDD with anhedonia.

The primary disturbed brain regions in MDD (either with or without anhedonia) in the current study include the CG, INS, and PrG. Large-scale meta-analyses have demonstrated a relationship between MDD and structural and functional abnormalities in these regions [49–51]. The current study found increased GMV–ALFF coupling in MDD patients in the right dorsal anterior CG, a crucial component of the DMN that plays a pivotal role in cognitive processes and mood regulation [52, 53]. Single-mode MRI studies have identified a reduction in the volume of the anterior CG and hypoactivation associated with MDD [49, 54]. Previous multimodal magnetic resonance studies have shown volume atrophy of the anterior CG in patients with MDD, as well as a negative correlation between global connectivity of the anterior CG and depressive symptoms [55, 56]. This finding is consistent with the current findings and suggests simultaneous alterations in CG structure and function in MDD. In addition, the present study revealed that GMV–ALFF coupling of the CG was a key predictor to distinguish MDD without anhedonia from HCs, suggesting asynchronous alterations in CG structure and function as a potential biological mechanism for MDD. Conversely, the GMV–ALFF decoupling was observed in the ventral dysgranular and granular INS, associated with interoception and sensory perception [57]. The INS acts as an interface between sensation, emotion, and cognition [58]. Hyperactivation of the INS and its reduced GMV and cortical thickness in relation to MDD have been independently reported in numerous studies [59–62]. A recent multimodal MRI study of structural–functional connectivity coupling suggested that in patients with MDD, functional communication in the INS cortex was tethered by anatomical pathways [63]. Thus, asynchronous alterations in INS structure and function may underlie the biology of MDD.

Notably, the current study found that bilateral PrGs exhibited GMV–ALFF decoupling in both groups of MDD patients compared to HC, whereas only the right side further exhibited decoupling in patients with MDD with anhedonia compared to MDD without anhedonia. The PrG is regarded as a critical part of the sensorimotor network [64]. The event-related potential, which instructs the intention to make a motor response, has generators in the primary motor cortex and premotor cortex and is modulated by depression [65, 66]. Previous studies have demonstrated increased PrG volume [67, 68] and decreased neural activity in MDD patients [16, 69]. Intriguingly, reduced activity in the PrG was observed in MDD during consummatory reward processes, suggesting its potential role in anhedonic symptoms [70]. The current study also demonstrated that the right PrG performs best in distinguishing MDD with anhedonia from HCs. Taken together, damage to PrG structure and function may be the biological mechanism for the formation of MDD and MDD with anhedonia.

In addition, the current study found that the right Tha, left MTG, and STG were more affected in the MDD with anhedonia group than in the other two groups, whereas there were no significant differences between the MDD without anhedonia group and the control group. The Tha interfaces with the reward circuitry of the brain, receiving input signals from the prefrontal cortex and outputting them to areas of the limbic system involved in emotional and reward processing [71, 72]. Increased regional neurophysiological activity requires higher metabolic demands, which were met through increased regional blood flow [73]. Evidence suggested that cerebral blood flow velocity was positively correlated with Tha volume in remitted MDD, whereas no association between Tha volume and cerebral blood flow was found in MDD with anhedonia [21]. Furthermore, MDD patients showed decreased activity in the right Tha during both reward anticipation and learning [74], and this was confirmed by the finding of decreased ALFF values in the right Tha in obsessive–compulsive disorder patients with anhedonia [75]. As such, previous studies and current findings consistently suggest a lack of synchronization of Tha structural and functional changes in MDD with anhedonia.

Regarding the temporal lobe, the current study observed that strong GMV–ALFF coupling was demonstrated in the left STG, whereas decoupling was demonstrated in the left MTG. The STG and MTG were not only involved in memory but also in the regulation of emotional information and cognition [76–80]. Not only has reduced temporal lobe volume been reported in patients with MDD in previous studies [81, 82], but reduced STG/MTG functional connectivity and regional activity have also been reported [83–85]. Furthermore, evidence indicating reduced STG activation and volume was considered to potentially underlie abnormal decision-making and reward-based learning in MDD patients [86–88]. Therefore, abnormalities in GMV–ALFF coupling in the temporal lobe may be associated with MDD with anhedonia.

Concurrently, this study revealed that the combined indices of the aforementioned distinct brain regions exhibited the most pronounced diagnostic utility in differentiating between MDD patients with and without anhedonia. This indicated that MDD was not attributable to structural or functional alterations in a single brain region but rather constituted the consequence of intricate interactions between the structure and function of multiple brain regions. This finding underscores the significance of multimodal fusion magnetic resonance studies in elucidating the biological mechanisms underlying MDD.

In this exploratory study, several limitations need to be noticed. From a methodological point of view, the sample size of the study was small, and the findings may not be generalizable. Moreover, this study employed a cross-sectional approach, thereby precluding an investigation of the causal relationship between structure-function coupling in brain regions and MDD with anhedonia. Longitudinal studies with large samples are needed to further explore the relationship between MDD with anhedonia and structure-function coupling in the future. Additionally, it is widely acknowledged that brain structure and function are dynamically changing, and current research has been limited to the exploration of structure-function coupling in the resting state of the brain at a certain moment in time, with a lack of exploration of dynamic changes in brain structure-function coupling.

## 5. Conclusion

The findings of the present cross-sectional study highlight the critical role of altered structure–function synchrony in the frontal lobe, temporal lobe, and Tha in MDD with anhedonia. In addition, structural–functional coupling abnormalities in the right PrG, right CG, and left MTG had strong diagnostic and predictive power, suggesting that these metrics may be reliable neuroimaging biomarkers individually or in combination to differentiate HCs and MDD with and without anhedonia. These insights provide an excellent avenue for developing targeted diagnostic and therapeutic strategies.

## Figures and Tables

**Table 1:** Demographic and clinical characteristics for all subjects (*n* = 92).

Variables	MDD with anhedonia, *n* = 29, mean (SD)	MDD without anhedonia, *n* = 33, mean (SD)	HCs, *n* = 30, mean (SD)	Analysis *F/t/χ*^2^	*p*-Values
Age (years)	28.48 (6.99)	30.73 (7.06)	27.20 (6.40)	2.165	0.121
Gender (male/female)	6/23	9/24	14/16	2.579	0.081
Education years	13.97 (3.05)	14.85 (2.18)	15.07 (2.32)	1.571	0.214
Onset age (years)	26.21 (6.75)	28.81 (7.82)	/	1.368	0.177
Episode times	1.67 (1.07)	1.47 (0.63)	/	0.868	0.389
Illness duration (months)	21.19 (20.61)	18.82 (23.30)	/	0.414	0.680
SHAPS score	38.14 (3.97)	26.94 (5.03)	/	9.642	<0.001
HAMD score	25.34 (3.24)	24.27 (3.84)	/	1.178	0.243
mFD	0.15 (0.12)	0.10 (0.05)	0.11 (0.05)	3.873	0.024

Abbreviations: HAMD, Hamilton Depression Scale; HCs, healthy controls; MDD, major depressive disorder; mFD, mean framewise displacement; SD, standard deviation; SHAPS, Snaith–Hamilton Pleasure Scale.

**Table 2:** Comparisons of the GMV–ALFF coupling of the brain regions among three groups.

Regions		Anatomical and modified Cyto-architectonic descriptions	MNI coordinates of peak voxel			*F*-values	*p*-Values	Adjusted *p* (FDR corrected)	*η* ^2^
			*X*	*Y*	*Z*				
Frontal lobe	PrG_R_6_4	A4t, area 4 (trunk region)	15	−22	71	10.044	<0.001	0.007	0.195
	PrG_L_6_3	A4ul, area 4 (upper limb region)	−26	−25	63	11.047	<0.001	0.006	0.210
Temporal lobe	STG_L_6_2	A41/42, area 41/42	−54	−32	12	6.212	0.003	0.046	0.130
	MTG_L_4_4	aSTS, anterior superior temporal sulcus	−58	−20	−9	9.340	<0.001	0.008	0.184
Insular lobe	INS_R_6_4	vId/vIg, ventral dysgranular and granular insula	39	−2	−9	7.686	<0.001	0.018	0.156
Limbic lobe	CG_R_7_1	A23d, dorsal area 23	4	−37	32	6.491	0.002	0.043	0.135
Subcortical nuclei	Tha_R_8_1	mPFtha, medial prefrontal thalamus	7	−11	6	7.972	<0.001	0.018	0.161

Abbreviations: *η*2, effect sizes; CG, cingulate gyrus; FDR, false discovery rate; INS, insular gyrus; MDD, major depressive disorder; MNI, Montreal Neurological Institute; MTG, middle temporal gyrus; PrG, precentral gyrus; STG, superior temporal gyrus; Tha, thalamus.

**Table 3:** The statistics of ROC curve analysis.

Regions	AUC	SEN	SPE	*p* Value	95% CI
A: MDD with anhedonia (*n* = 29) and HC (*n* = 30)					
PRE.A	0.993	100%	93.33%	<0.001	0.926–1.000
A4ul.L	0.799	79.31%	66.67%	<0.001	0.674–0.892
aSTS.L	0.700	58.62%	76.67%	0.004	0.567–0.812
A41/42.L	0.774	96.55%	56.67%	<0.001	0.646–0.872
A4t.R	0.806	86.21%	73.33%	<0.001	0.682–0.897
vId/vIg.R	0.689	75.86%	73.33%	0.009	0.555–0.803
A23d.R	0.628	62.07%	76.67%	0.012	0.547–0.797
mPFtha.R	0.747	58.62%	83.33%	<0.001	0.617–0.851
B: MDD without anhedonia (*n* = 33) and HC (*n* = 30)					
PRE.B	0.923	81.82%	96.67%	<0.001	0.828−0.975
A4ul.L	0.764	66.67%	83.33%	<0.001	0.640–0.862
A4t.R	0.691	63.64%	73.33%	0.005	0.562 −0.801
vId/vIg.R	0.738	78.79%	73.33%	<0.001	0.612 −0.841
A23d.R	0.776	60.61%	90%	<0.001	0.653–0.871
C: MDD with anhedonia (*n* = 29) and MDD without anhedonia (*n* = 33)					
PRE.C	0.866	72.41%	90.91	<0.001	0.756 −0.939
aSTS.L	0.774	58.62%	87.88%	<0.001	0.650–0.871
A41/42.L	0.721	96.55%	45.45%	<0.001	0.593–0.827
A4t.R	0.651	68.97%	60.61%	0.033	0.519–0.768
mPFtha.R	0.649	51.72%	81.82%	0.040	0.517–0.766

Abbreviations: A23d, dorsal area 23; A41/42, area 41/42; A4t, area 4 (trunk region); A4ul, area 4 (upper limb region); aSTS, anterior superior temporal sulcus; AUC, area under the ROC curve; CIs, confidence intervals; MDD, major depressive disorder; mPFtha, medial prefrontal thalamus; PRE, prediction probability *p*; SEN, sensitivity; SPE, specificity; vId/vIg, ventral dysgranular and granular insula.

## Data Availability

The datasets generated and/or analyzed during the current study are not publicly available due to privacy and ethical restrictions but are available from the corresponding author upon reasonable request.
